# Effects of Different *Eimeria* Inoculation Doses on Growth Performance, Daily Feed Intake, Gut Health, Gut Microbiota, Foot Pad Dermatitis, and *Eimeria* Gene Expression in Broilers Raised in Floor Pens for 35 Days

**DOI:** 10.3390/ani13132237

**Published:** 2023-07-07

**Authors:** Janghan Choi, Doyun Goo, Milan Kumar Sharma, Hanseo Ko, Guanchen Liu, Deependra Paneru, Venkata Sesha Reddy Choppa, Jihwan Lee, Woo Kyun Kim

**Affiliations:** 1 Department of Poultry Science, University of Georgia, Athens, GA 30602, USA; choij@uga.edu (J.C.); dgoo@uga.edu (D.G.); milan.sharma@uga.edu (M.K.S.); hsko@uga.edu (H.K.); guanchen.liu@uga.edu (G.L.); dpaneru@uga.edu (D.P.); venkataseshareddy.choppa@uga.edu (V.S.R.C.); jihwan.lee@uga.edu (J.L.); 2 US National Poultry Research Center, United States Department of Agriculture Agricultural Research Service, Athens, GA 30605, USA

**Keywords:** *Eimeria*, apparent ileal digestibility, gut microbiota, body composition, floor pen, broilers

## Abstract

**Simple Summary:**

Coccidiosis, which is induced by *Eimeria* spp., is one of the most predominant diseases and causes tremendous economic losses in the world. The effects of *Eimeria* infection on broilers at slaughter ages in floor pen conditions should be elucidated to conduct further studies investigating the effects of feed additives and bioactive compounds as alternatives for anti-coccidial drugs in broilers. The study was aimed to investigate the effects of different inoculation doses of *E. acervulina*, *E. maxima*, and *E. tenella* with different doses on growth performance, gut ecosystem, and oocyst shedding in broilers raised in floor pens for 35 days. *Eimeria* infection decreased body weight (BW) in the acute phase (D 21), and this effect was prolonged to the final day (D 35). *Eimeria* oocysts were observed in the litter until D 35, which may indicate that *Eimeria* spp. reinfected broilers. *Eimeria* infection dramatically reduced crude fat (CF) digestibility in the acute phase, which may be associated with reduced fat content in the broilers on D 35. Gut microbiota was negatively affected by *Eimeria* infection in both acute phase and on D 35. In conclusion, *Eimeria* infection negatively affected the growth performance and gut ecosystem in broilers, and the negative effects were prolonged to D 35 in floor pen conditions.

**Abstract:**

The study was conducted to investigate the effects of different *Eimeria* inoculation doses on the growth performance, gut ecosystem, and body composition of broilers in floor pens for 35 days. A total of 750 15-day-old broilers were allocated to five experimental groups with six replicate pens. The five experimental groups included unchallenged control (CON); *Eimeria* dose 1 (ED1): *E. acervulina*: 31,250/*E. maxima*: 6250/*E. tenella*: 6250; *Eimeria* dose 2 (ED2): *E. acervulina*: 62,500/*E. maxima*: 12,500/*E. tenella*: 12,500; *Eimeria* dose 3 (ED3): *E. acervulina*: 125,000/*E. maxima*: 25,000/*E. tenella*: 25,000; and *Eimeria* dose 4 (ED4): *E. acervulina*: 250,000/*E. maxima*: 50,000/*E. tenella*: 50,000. On D 21, BW were linearly reduced by increased *Eimeria* inoculation doses (*p* < 0.01). On D 35, the *Eimeria* challenge groups had significantly lower BW compared to the CON group. Increased *Eimeria* inoculation doses linearly decreased crude fat (CF) (*p* < 0.01) on D 21. Increased *Eimeria* inoculation doses tended to increase the relative abundance of the phylum Proteobacteria (*p* = 0.098) on D 21. On D 35, lean:fat was linearly reduced by increased *Eimeria* inoculation doses (*p* < 0.05). *Eimeria* infection negatively influenced growth performance and gut health in broilers in the acute phase, and the negative effects were prolonged to D 35 in floor pen conditions.

## 1. Introduction

Coccidiosis, which is induced by *Eimeria* spp., is one of the most predominant diseases and causes tremendous economic losses estimated to be approximately USD 3 billion to 10.4 billion pounds annually in worldwide broiler production [1]. There are seven known *Eimeria* spp. in chickens, and *Eimeria acervulina*, *E. tenella*, and *E. maxima* are known to be most predominant *Eimeria* spp. in broiler production [2]. The *Eimeria* life cycle contains the exogenous (e.g., sporulation in the excreta) and the endogenous (asexual and sexual reproduction in the host intestine) life cycles [3]. During the endogenous phase, the activities of *Eimeria* spp. disrupt the functionality and integrity of the gastrointestinal tract of chickens, which induces watery feces and negatively influences nutrient utilization, growth performance, and animal welfare in the acute phase [4,5]. Watery feces can increase the moisture content of the litter, which can increase the incidence and severity of foot pad dermatitis (FPD) by increasing the litter ammonia level [6]. Decreased nutrient digestion and absorption due to *Eimeria* infection in the acute phase have a potential to alter body composition of broiler chickens at slaughter ages [7].

Traditionally, anti-coccidial drugs such as ionophores and synthetic drugs have been utilized for the control of coccidiosis in broilers [8]. However, due to public concerns regarding the spread of antibiotic-resistant microbes, the utilization of anti-coccidial drugs has been restricted in many countries [9]. Therefore, it has become essential to explore alternatives to anti-coccidial drugs in poultry production. Diverse bioactive compounds including amino acids [10], essential oils [11], and plant extracts [12] were studied to use as alternatives for anticoccidial drugs in broiler production. However, most of the *Eimeria* studies were conducted in cage conditions. *Eimeria* trials conducted in cage conditions have following limitations: (1) reinfection of *Eimeria* via the oral–fecal route is unlikely to occur because feces are regularly removed before they can build up in cages; (2) some cages are not suitable for accommodating broilers during the finisher phase (D 28 to 42); and (3) cage conditions cannot completely mimic the field conditions because broilers are normally raised in the litter condition in the poultry industry [13]. Proper research settings for *Eimeria* studies in floor pens are required, and the effects of *Eimeria* infection with different inoculation doses on growth performance, gut health, gut microbiota, and body composition in broilers at slaughter ages in the floor pen conditions should be elucidated to conduct further studies investigating the effects of feed additives and bioactive compounds as alternatives for anti-coccidial drugs in broilers. Therefore, this study was designed to investigate the effects of different *Eimeria* inoculation doses on the growth performance, litter moisture content, oocyst shedding, *Eimeria* gene expression, nutrient digestion and absorption, gut microbiota, incidence and severity of FPD, and body composition in broilers raised in floor pens for 35 days.

## 2. Materials and Methods

### 2.1. Experimental Design, Animals, Experimental Infection, Diets, and Growth Performance

The Institutional Animal Care and Use Committee at the University of Georgia approved animal care and use protocols of the current study. A total of 750 fifteen-day-old Cobb 500 male broilers were randomly distributed to 5 experimental groups with 6 replicate pens of 25 birds per pen. The 5 experimental groups were different inoculation doses of *E. acervulina*, *E. maxima*, and *E. tenella* as follows: unchallenged control (CON); *Eimeria* dose 1 (ED1): *E. acervulina*: 31,250/*E. maxima*: 6250/*E. tenella*: 6250; *Eimeria* dose 2 (ED2): *E. acervulina*: 62,500/*E. maxima*: 12,500/*E. tenella*: 12,500; *Eimeria* dose 3 (ED3): *E. acervulina*: 125,000/*E. maxima*: 25,000/*E. tenella*: 25,000; and *Eimeria* dose 4 (ED4): *E. acervulina*: 250,000/*E. maxima*: 50,000/*E. tenella*: 50,000. One milliliter of PBS containing freshly prepared *Eimeria* spp. (stored in the 5% potassium dichromate solution at 4 °C for less than 3 months) was administered to individual birds on D 15 [12]. The doses for *Eimeria* spp. were determined according to our previous study [14]. Birds were reared in floor pens (width: 1.52 m, length: 1.22 m, height: 0.61 m) equipped with one feeder and several nipple drinkers, and birds had free access to water and feed during the entire experimental period. The front side was blocked by wire, while the sides are blocked by hardboard, indicating the front side was more vulnerable for cross contamination. The CON group was distributed in a way that it could be located across from the front side to minimize the cross contamination, and *Eimeria* infected groups were randomly allocated. Temperature and light were managed according to Cobb 500 broiler management guide (2018). Diets were formulated to meet or exceed the recommended level according to Cobb 500 nutrient requirement guide (2018) and contained 0.3% titanium dioxide (Acros Organics, Morris Plains, NJ, USA) as an inert marker as shown in Table 1. The diets for the CON group in all phases contained 0.05% anticoccidial drug (Coban 90, Elanco Animal Health, Greenfield, IN, USA) to prevent cross contamination to the CON group. The feeding phases were divided into the grower (D 15 to 28) and finisher phases (D 28 to 35). Growth performance parameters including body weight (BW), average daily gain (ADG), average daily feed intake (ADFI), and feed conversion ratio (FCR) were measured on D 21 (6 days post inoculation (dpi)), D 28, and D 35, and daily feed intake (DFI) was measured through the entire experiment period.

### 2.2. Lesion Score, Oocyst Shedding, Gut Permeability, and Digesta and Litter Moisture Content

On D 21 (6 dpi), D 28, and D 35, duodenum, jejunum-ileum section, and ceca were collected, and lesion scores for each section were evaluated according to the 4-score scale [15]. On 22 (7 dpi), D 28, and D 35, cloaca content from one sacrificed bird per pen and approximately 30 g of litter samples were collected for oocyst shedding. Litter samples were collected in the different areas of the pen except areas near water nipples, and the litter samples were thoroughly mixed. Tap water (40 mL) were added to the samples (5 g), and the samples were placed at room temperature overnight to dissolve hard fecal particles. Afterwards, the samples were vortexed and diluted 10 times with saturated salt solution. *E. acervulina*, *E. maxima*, and *E. tenella* in the solution were counted using a hemocytometer (Hausser Scientific Company, Horsham, PA, USA). Ileal content and litter samples were oven-dried (75 °C) until a constant weight was achieved to determine their moisture content [16]. Gut permeability by using fluorescein isothiocyanate–dextran (molecular weight: 4 kDa; FITC-D4; Sigma-Aldrich Co., St. Louis, MO, USA) was determined on D 20 (5 dpi) and D 27 according to Choi et al. [12]. The concentration of FITD-F4 in the serum was determined using a prepared standard curve.

### 2.3. Foot Pad Dermatitis (FPD) and Body Composition Analysis

On D 35, severity and incidence of FPD were recorded according to Sorin et al. [17], as shown in Table 2.

**Figure 1:**
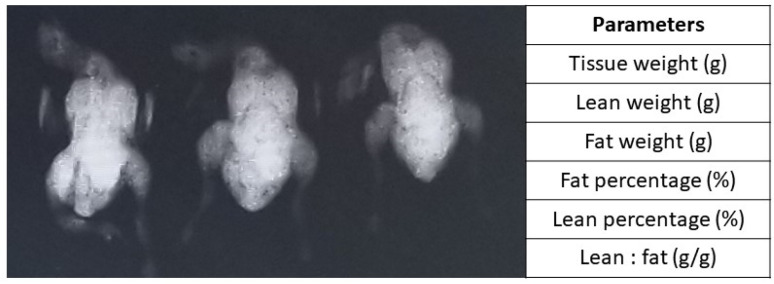
Scanned images of dual-energy X-ray absorptiometry (DEXA) of broiler chickens and corresponding parameters.

One bird per pen was euthanized via cervical dislocation and scanned using a dual-energy X-ray absorptiometry (DEXA, GE Healthcare, Madison, WI, USA) as shown in Figure 1.

### 2.4. Apparent Ileal Digestibility (AID) of Nutrients

On D 21 (6 dpi) and D 35, ileal content was collected between 10 cm below of the Meckel’s diverticulum and 10 cm upper of the ileo-ceca-colonic junction. Feed (0.5 g) and ileal samples (0.3 g) were ashed at 600 °C overnight, and concentrations of titanium dioxide were analyzed according to Short et al. [18]. The concentrations of CP and crude fat (CF) were determined using nitrogen combustion analyses according to AOAC international (2000) analytical method 990.03 and analytical method 942.05, respectively. Apparent ileal digestibility (AID) of dry matter (DM), organic matter (OM), ash, CP, and CF were calculated according to Lin and Olukosi [19].

### 2.5. Intestinal Morphology

On D 21 (6 dpi) and D 35, the duodenum (mid-part of the duodenal loop), jejunum (10 cm upper of the Meckel’s diverticulum), and mid-ceca were collected, and remaining digesta was rinsed with PBS and placed into 10% neutral buffered formalin solution. After 72 h fixation, samples were embedded in paraffin and sliced into 4 µm sections, and hematoxylin and eosin (H&E) staining was performed. The stained slides were pictured using a microscope (BZ-X810; Keyence, Osaka, Japan). The villus height (VH) and crypt depth (CD) were measured for the duodenum and jejunum samples, and CD was measured for the ceca samples using ImageJ (National Institutes of Health, Bethesda, MD, USA).

### 2.6. Activities of Jejunal Brush Border Digestive Enzymes and Alkaline Phosphatase in the Serum

On D 21 (6 dpi) and D 35, the jejunum (located 10 cm above the Meckel’s diverticulum) was collected and immediately snap-frozen in liquid nitrogen. It was then stored at −80 °C for further analysis. Around 100 mg of the jejunum samples (10 cm upper of the Meckel’s diverticulum) were homogenized in 1.8 mL PBS by using a beads beater (Biospec Products, Bartlesville, OK, USA). The homogenized samples were centrifuged at 12,000× *g* and 4 °C for 15 min, and the protein concentration of the supernatant was determined using Pierce BCA Protein Assay Kits (Thermo Fisher Scientific, Waltham, MA, USA). Activities of maltase and sucrase were evaluated according to Fan et al. [20]. Activities of alkaline phosphatase in the intestine and serum were determined according to Lackeyram et al. [21]. Activities of aminopeptidase N (APN) were measured according to the method by Maroux et al. [22]. Lipase activities were analyzed according to the method of Elgharbawy et al. [23]. The activities of digestive enzymes were expressed as values per mg protein.

### 2.7. RNA Extraction and Quantitative Real-Time Reverse Transcription PCR (qRT-PCR)

On D 21 (6 dpi), whole-tissue samples of the duodenum (mid-part of the duodenal loop), jejunum (10 cm upper of the Meckel’s diverticulum), and mid-ceca were collected and immediately snap-frozen in liquid nitrogen. They were then stored at −80 °C for further analysis. Around 100 mg of the whole-tissue of the duodenum (mid-part of the duodenal loop), jejunum (10 cm upper of the Meckel’s diverticulum), and mid-ceca was homogenized in QIAzol lysis reagents (Qiagen, Valencia, CA, USA) using a beads beater (Biospec Products, Bartlesville, OK, USA). RNA was extracted according to the manufacturer’s procedure, and RNA quantity was measured using a NanoDrop 2000 spectrophotometer (Thermo Fisher Scientific, Waltham, MA, USA). One microgram of RNA was utilized to synthesize the first-strand cDNA using high-capacity cDNA synthesis kits (Applied Biosystems, Foster City, CA, USA). Primers used in the study are presented in Table 3.

Quantitative real-time reverse transcription PCR (qRT-PCR) was conducted using SYBR Green Master Mix with a Step One thermocycler (Applied Biosystems, Foster City, CA, USA). The final PCR volume (10 μL) contained 5 μL of SYBR Green Master Mix, 1.5 μL of cDNA, 0.5 μL of forward and reverse primers (10 μM), and 2.5 μL of water. Thermal cycle conditions for all reactions were as follows: 95 °C denature for 10 min, 40 cycles at 95 °C for 15 s and 60 °C for 1 min, 95 °C for 15 s, 60 °C for 1 min and 95 °C for 15 s. The melting curve of each gene was checked to confirm the specificity of each PCR product. Several PCR products from each gene were stained with 6 × DNA loading dye (Thermo Fisher Scientific, Waltham, MA, USA), electrophoresed on a 3% agarose gel in a Tris-acetate-EDTA buffer, and visualized by adding ethidium bromide to confirm the specificity of each PCR product. Relative abundance of *Eimeria 18s* genes was normalized by using host reference genes (geometric mean of beta actin and glyceraldehyde 3-phosphate dehydrogenase) to quantify *Eimeria* spp. in the host tissue, and relative abundance of *Eimeria* genes was normalized with a housekeeping gene (18s) for each *Eimeria* spp. [24]. Relative mRNA abundance was determined by using the 2^−∆∆Ct^ method [25]. The negative control, containing no cDNA, was included in each run, and each sample was run in duplicate.

### 2.8. DNA Extraction and Microbiome Analysis

On D 21 (6 dpi) and D 35, the cecal content was collected and immediately snap-frozen in liquid nitrogen. It was then stored at −80 °C for further analysis. DNA was extracted from the cecal content by employing QIAamp^®^ DNA stool mini kit (Qiagen GmbH, Hilden, Germany) according to manufacturer’s protocol. Quality and quantity of extracted DNA were checked using a NanoDrop 2000 spectrophotometer (Thermo Fisher Scientific), and samples were shipped to LC sciences (Houston, TX, USA) for 16s rRNA gene sequencing. Qimme2 (version 2022.02) was used to process and analyze 16s rRNA gene sequences [26]. Using Qiime2’s built-in functions, alpha diversity, beta diversity, and the phylum and family level composition were analyzed and presented.

### 2.9. Statistical Analyses

Statistical analyses and graph construction were performed using SAS (version 9.4; SAS Inst. Inc., Cary, NC, USA) and GraphPad Prism (Version 9.1.0; GraphPad Software, San Diego, CA, USA), respectively. All experimental groups were compared using PROC MIXED in a completely randomized design followed by the Tukey’s HSD (honestly significant difference) test. For quantitative beta diversity measurement, each experimental group was placed as the control group, and experimental groups were compared using PROC MIXED with Dunnett’s post hoc test. *Eimeria* lesion score data were compared using Kruskal–Wallis test followed by the Dwass–Steel–Critchlow–Fligner test. Orthogonal polynomial contrasts were performed to analyze the significance of linear or quadratic effects of different *Eimeria* doses, and the inoculation doses of *E. acervulina*, *E. maxima*, and *E. tenella* were normalized by using the base 2 logarithm of the number of sporulated Eimeria oocyst number for orthogonal polynomial contrasts. Statistical significance was set at *p* < 0.05, and trends (0.05 ≤ *p* ≤ 0.1) were also shown.

## 3. Results

### 3.1. Growth Performance and Daily Feed Intake (DFI)

The results of the growth performance are shown in Table 4. On D 21, BW was linearly (*p* < 0.01) and quadratically (*p* < 0.05) decreased due to increased *Eimeria* inoculation doses, the ED1 and ED2 groups had significantly lower BW compared to the CON group, and the ED4 group had significantly lower BW compared to the ED1 and ED2 groups. Increased *Eimeria* inoculation doses linearly and quadratically reduced ADFI (*p* < 0.01), and the CON group and the ED4 group had the highest and lowest ADFI among the experimental groups, respectively (*p* < 0.05). The FCR was linearly increased by increased *Eimeria* inoculation doses (*p* < 0.01), and the ED4 group and the CON group had the highest and lowest FCR, respectively, among the experimental groups (*p* < 0.01).

On D 28, increased *Eimeria* inoculation doses linearly (*p* < 0.01) and quadratically (*p <* 0.05) decreased BW, ADG, and FCR, and the *Eimeria* challenge groups had lower BW, ADG, and ADFI compared to the CON group. On D 35, *Eimeria* challenged groups had significantly lower BW compared to the CON group, and increased *Eimeria* doses linearly (*p* < 0.05) and quadratically (*p* < 0.05) reduced BW. In the whole phase, increased *Eimeria* inoculation doses linearly (*p* < 0.01) and quadratically (*p* < 0.01) reduced ADG, and the *Eimeria* infected groups had lower ADG compared to the CON group (*p* < 0.01). The ADFI were linearly (*p* < 0.01) and quadratically (tendency; *p* = 0.076) decreased by *Eimeria* infection, and the *Eimeria* challenged groups had lower ADFI compared to the CON group in the whole phase (*p* < 0.01). The CON group and the ED4 group had the lowest and highest FCR, respectively, among the experimental groups (*p* < 0.01), and increased *Eimeria* inoculation doses resulted in a linear increase in FCR in the whole phase (*p* < 0.01).

**Figure 2:**
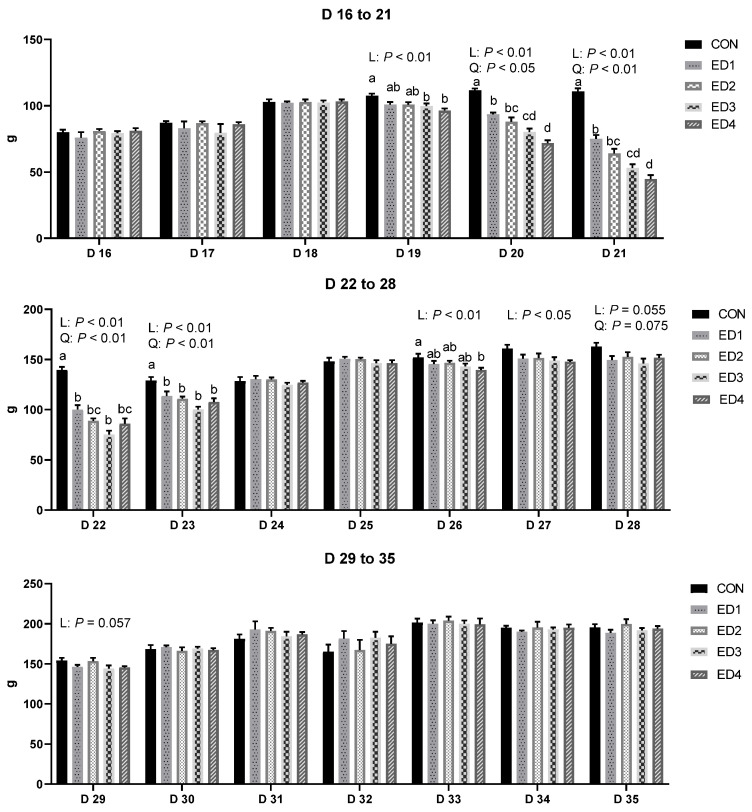
Daily feed intake (g) during the entire experimental period in the unchallenged control (CON); *Eimeria* dose 1 (ED1): *E. acervulina*: 31,250/*E. maxima*: 6250/*E. tenella*: 6250; *Eimeria* dose 2 (ED2): *E. acervulina*: 62,500/*E. maxima*: 12,500/*E. tenella*: 12,500; *Eimeria* dose 3 (ED3): *E. acervulina*: 125,000/*E. maxima*: 25,000/*E. tenella*: 25,000; and *Eimeria* dose 4 (ED4): *E. acervulina*: 250,000/*E. maxima*: 50,000/*E. tenella*: 50,000 groups. Oral gavage of *Eimeria* spp. was performed on D 15. Experimental groups were compared using PROC MIXED followed by the Tukey’s HSD (honestly significant difference) test, and different letters mean significant differences (*p* < 0.05) among the experimental groups on the same day. Orthogonal polynomial contrasts were conducted to see linear and quadratic patterns among the experimental groups on the same day.

The DFI during the entire experimental period is presented in Figure 2. On D 19, DFI was linearly reduced by increased *Eimeria* inoculation doses (*p* < 0.01). From D 20 to 23, DFI was linearly (*p* < 0.01) and quadratically (*p* < 0.05) decreased by increased *Eimeria* inoculation doses. Increased *Eimeria* inoculation doses linearly decreased DFI on D 26 (*p* < 0.01) and D 27 (*p* < 0.05). On D 28, increased *Eimeria* inoculation doses tended to linearly (*p* = 0.055) and quadratically (*p* = 0.075) reduce DFI. On D 29, the *Eimeria* challenge tended to linearly reduce DFI (*p* = 0.057). No differences were observed in the DFI from D 30 to 35 (*p* > 0.1).

### 3.2. Lesion Score and Gut Permeability

**Figure 3:**
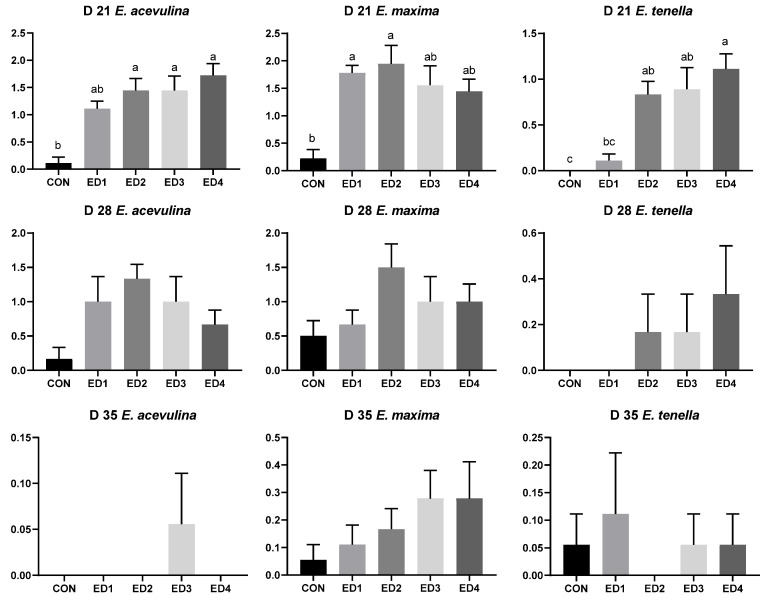
Duodenal, jejunal, and cecal lesion score in the unchallenged control (CON); *Eimeria* dose 1 (ED1): *E. acervulina*: 31,250/*E. maxima*: 6250/*E. tenella*: 6250; *Eimeria* dose 2 (ED2): *E. acervulina*: 62,500/*E. maxima*: 12,500/*E. tenella*: 12,500; *Eimeria* dose 3 (ED3): *E. acervulina*: 125,000/*E. maxima*: 25,000/*E. tenella*: 25,000; and *Eimeria* dose 4 (ED4): *E. acervulina*: 250,000/*E. maxima*: 50,000/*E. tenella*: 50,000 groups on D 21, D 28, and D 35. Oral gavage of *Eimeria* spp. was performed on D 15. Lesion scores for each section were evaluated according to the 4-score scale [15]. Experimental groups were compared using Kruskal–Wallis test followed by the Dwass–Steel–Critchlow–Fligner test. Different letters mean significant differences (*p* < 0.05) among the experimental groups.

On D 21, the CON group had significantly lower lesion scores for *E. acervulina*, *E. maxima*, and *E. tenella* compared to the Eimeria infected groups as shown in Figure 3. The ED4 group had significantly higher *E. tenella* lesion scores compared to the ED1 group. On D 28, although no statistical differences in *E. acervulina*, *E. maxima*, and *E. tenella* lesion scores were observed among the experimental groups (*p* > 0.1), lesion scores of *E. acervulina* and *E. maxima* were observed in all experimental groups, and *E. tenella* lesion score was observed in the ED2, ED3, and ED4 groups. On D 35, *E. acervulina* lesion scores were observed only in the ED3 group, and *E. maxima* lesion scores were observed in all experimental groups. *E. tenella* lesion scores were observed in all experimental groups except the ED2 group.

As shown in Table 5, on D 20, the ED4 group had significantly higher gut permeability compared to the CON and ED1 groups, and the ED3 group had significantly higher gut permeability compared to the ED2 group. However, no significant differences were observed in gut permeability on D 27 (*p* > 0.1).

### 3.3. Digesta and Litter Moisture Content and Foot Pad Dermatitis (FPD)

As shown in Table 6, on D 21 and D 35, increased *Eimeria* inoculation doses resulted in a linear increase in ileal moisture content (*p* < 0.01), and the ED4 group had significantly higher ileal moisture content compared to the CON group. On D 21, litter moisture content tended to be increased in a linear trend by increased *Eimeria* inoculation doses (*p* = 0.098). Increased *Eimeria* inoculation doses caused a quadratic increase in the litter moisture content on D 35 (*p* < 0.05).

**Figure 4:**
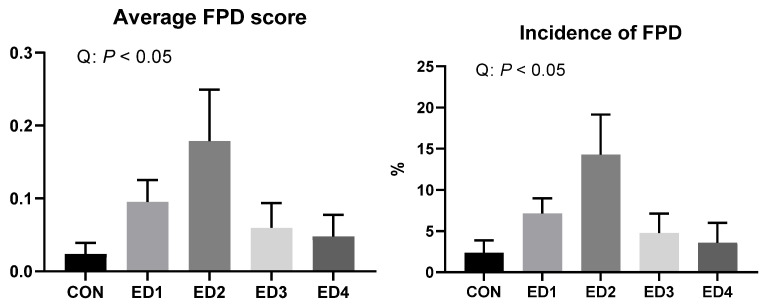
Foot pad dermatitis (FPD) in the unchallenged control (CON); *Eimeria* dose 1 (ED1): *E. acervulina*: 31,250/*E. maxima*: 6250/*E. tenella*: 6250; *Eimeria* dose 2 (ED2): *E. acervulina*: 62,500/*E. maxima*: 12,500/*E. tenella*: 12,500; *Eimeria* dose 3 (ED3): *E. acervulina*: 125,000/*E. maxima*: 25,000/*E. tenella*: 25,000; and *Eimeria* dose 4 (ED4): *E. acervulina*: 250,000/*E. maxima*: 50,000/*E. tenella*: 50,000 groups on D 35. Oral gavage of *Eimeria* spp. inoculum (1 mL of PBS) was performed on D 15. Severity (0 to 2), and incidence of FPD was recorded according to Sorin et al. [17]. Experimental groups were compared using Kruskal–Wallis test followed by the Dwass–Steel–Critchlow–Fligner test. Orthogonal polynomial contrasts were conducted to see linear and quadratic patterns among the experimental groups.

The severity and incidence of FPD on D 35 were quadratically increased by increased *Eimeria* inoculation doses (*p* < 0.05; Figure 4).

### 3.4. Oocyst Shedding

Table 7 shows that increased inoculation doses of *E. acervulina*, *E. maxima*, and *E. tenella* linearly increased the number of oocysts of *E. acervulina* and *E. tenella* (*p* < 0.05) and tended to linearly increase *E. maxima* oocyst in the cloaca content (*p* = 0.081) on D 22. The oocyst number of *E. maxima* in the cloaca were quadratically modulated by increased doses of *E. maxima* (*p* < 0.05). Linearly increased number of oocysts of *E. acervulina* and *E. tenella* in the litter were observed by increased infection doses of *Eimeria* spp. (*p* < 0.01), and the ED3 and ED4 groups had significantly higher *E. acervulina* oocysts in the litter compared to the ED1 group, and the ED2 and ED3 groups had significantly higher *E. tenella* oocysts compared to the ED1 group on D 22. However, no statistical differences were observed in the number of *Eimeria* oocysts among the *Eimeria* challenged groups (*p* > 0.1), *Eimeria* oocysts were observed either in the cloaca content and litter in the *Eimeria* infected groups on D 28 and 35.

### 3.5. Apparent Ileal Digestibility (AID) of Nutrients

As shown in Table 8, increased *Eimeria* inoculation doses linearly decreased the AID of CP and EE (*p* < 0.01) on D 21, and the AID of EE tended to be quadratically reduced by increased *Eimeria* inoculation doses (*p* = 0.056). The ED4 group had a significantly lower AID of CP compared to the CON group. The ED3 and ED4 groups had a significantly lower AID of EE compared to the CON group, and the ED2, ED3, and ED4 groups had minus values for the AID of CF. On D 35, the AID of ash was linearly decreased by increased *Eimeria* inoculation doses (*p* < 0.05).

### 3.6. Intestinal Morphology

As shown in Table 9, duodenal VH were linearly reduced by increased *Eimeria* inoculation doses (*p* < 0.01), and the ED3 and ED4 groups had significantly lower duodenal VH compared to the CON and ED1 groups. Duodenal CD was linearly deepened by increased *Eimeria* inoculation doses (*p* < 0.01), and the CON group had lower duodenal CD compared to the ED2, ED3, and ED4 groups (*p* < 0.01). Duodenal VH:CD were linearly reduced by increased *Eimeria* inoculation doses (*p* < 0.01), and the *Eimeria* challenged groups has significantly lower duodenal VH:CD compared to the CON group (*p* < 0.01). Jejunal VH was linearly decreased by increased *Eimeria* inoculation doses (*p* < 0.01), and the ED3 and ED4 groups had significantly lower jejunal VH compared to the CON and ED1 groups. Jejunal CD was linearly deepened by increased *Eimeria* inoculation doses (*p* < 0.01), and the CON group had the lowest jejunal CD among the experimental groups (*p* < 0.01). Jejunal VH:CD was linearly reduced by increased *Eimeria* inoculation doses (*p* < 0.01), and the ED3 and ED4 groups had the lowest jejunal VH:CD among the experimental groups (*p* < 0.01), and the CON group had the highest jejunal VH:CD among the experimental groups (*p* < 0.01). Cecal CD was linearly deepened by increased *Eimeria* inoculation doses (*p* < 0.01), and the ED3 and ED4 groups had significantly deeper cecal CD compared to the CON group (*p* < 0.05). On D 35, duodenal CD was quadratically deepened by increased *Eimeria* inoculation doses (*p* < 0.01), and the ED2 group had significantly higher duodenal CD compared to the CON group. Increased *Eimeria* inoculation doses resulted in a quadratic increase in duodenal VH:CD (*p* < 0.05).

### 3.7. Activities of Jejunal Brush Border Digestive Enzymes

As shown in Table 10, activities of APN were lower in the ED1 group compared to the CON group on D 21 (*p* < 0.05). Activities of serum alkaline phosphatase (SAP) were linearly reduced by increased *Eimeria* inoculation doses (*p* < 0.01), and the ED3 and ED4 groups had significantly lower activities of SAP compared to the CON group. The ED2 group had significantly higher sucrase activities compared to the ED3 group. Increased *Eimeria* inoculation doses linearly decreased maltase activities (*p* < 0.01), and the ED1 and ED4 groups had significantly lower maltase activities compared to the CON group. On D 35, activities of APN were linearly decreased by increased *Eimeria* infection doses (*p* < 0.05).

### 3.8. Alpha Diversity of the Cecal Microbiome Communities

No differences were observed in the alpha diversity parameters (biodiversity of the samples) on D 21 (*p* > 0.1; Table 11). On D 35, alpha diversity parameters including pielou evenness (evenness; *p* < 0.05), faith phylogenetic diversity (biodiversity based on phylogeny; *p* < 0.05), shannon entropy (richness and evenness; *p* < 0.05), and observed features (richness; *p* < 0.01) were linearly reduced by increased *Eimeria* inoculation doses.

### 3.9. Beta Diversity of the Cecal Microbiome Communities

**Figure 5:**
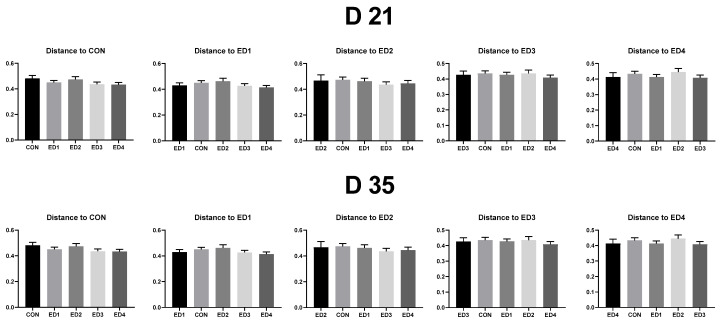
Unweighted unifrac measurement (quantitative beta diversity) in the unchallenged control (CON); *Eimeria* dose 1 (ED1): *E. acervulina*: 31,250/*E. maxima*: 6250/*E. tenella*: 6250; *Eimeria* dose 2 (ED2): *E. acervulina*: 62,500/*E. maxima*: 12,500/*E. tenella*: 12,500; *Eimeria* dose 3 (ED3): *E. acervulina*: 125,000/*E. maxima*: 25,000/*E. tenella*: 25,000; and *Eimeria* dose 4 (ED4): *E. acervulina*: 250,000/*E. maxima*: 50,000/*E. tenella*: 50,000 groups. groups. Oral gavage of *Eimeria* spp. inoculum (1 mL of PBS) was performed on D 15. Each experimental group was placed as the control group, and experimental groups were compared by using one-way PROC MIXED with Dunnett’s post hoc test.

As shown in Figure 5, no differences were observed in unweighted unifrac distance (dissimilarity among samples without considering abundance information) to each experimental group both on D 21 and D 35 (*p* > 0.1).

**Figure 6:**
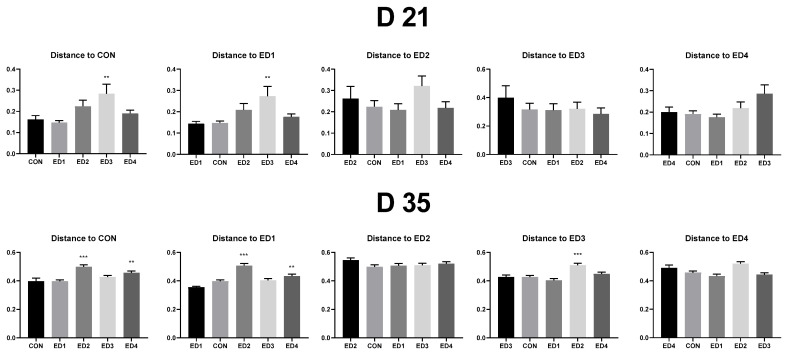
Weighted unifrac measurement (quantitative beta diversity) in the unchallenged control (CON); *Eimeria* dose 1 (ED1): *E. acervulina*: 31,250/*E. maxima*: 6250/*E. tenella*: 6250; *Eimeria* dose 2 (ED2): *E. acervulina*: 62,500/*E. maxima*: 12,500/*E. tenella*: 12,500; *Eimeria* dose 3 (ED3): *E. acervulina*: 125,000/*E. maxima*: 25,000/*E. tenella*: 25,000; and *Eimeria* dose 4 (ED4): *E. acervulina*: 250,000/*E. maxima*: 50,000/*E. tenella*: 50,000 groups. Oral gavage of *Eimeria* spp. inoculum (1 mL of PBS) was performed on D 15. Each experimental group was placed as the control group, and experimental groups were compared by using one-way PROC MIXED with Dunnett’s post hoc test. ** represents when *p* < 0.05, and *** represents when *p* < 0.01.

Figure 6 shows that on D 21, the ED3 group had significantly higher weighted unifrac distance (dissimilarity among samples with considering abundance information) to compared to the CON and ED1 groups (*p* < 0.05). On D 35, the ED2 (*p* < 0.01) and ED4 (*p* < 0.05) groups had significantly higher weighted unifrac distance compared to the CON and ED1 groups. The ED2 group had significantly higher weighted unifrac distance compared to the ED3 group (*p* < 0.01).

**Figure 7:**
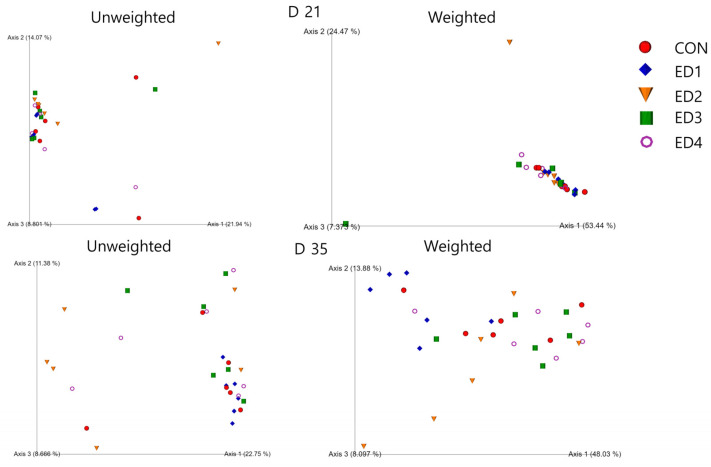
Visualized beta diversity indices including unweighted and weighted emperor in the unchallenged control (CON); *Eimeria* dose 1 (ED1): *E. acervulina*: 31,250/*E. maxima*: 6250/*E. tenella*: 6250; *Eimeria* dose 2 (ED2): *E. acervulina*: 62,500/*E. maxima*: 12,500/*E. tenella*: 12,500; *Eimeria* dose 3 (ED3): *E. acervulina*: 125,000/*E. maxima*: 25,000/*E. tenella*: 25,000; and *Eimeria* dose 4 (ED4): *E. acervulina*: 250,000/*E. maxima*: 50,000/*E. tenella*: 50,000 groups.

As shown in Figure 7, no visual differences were observed in beta diversity parameters including unweighted unifrac and weighted unifrac on D 21 and D 35.

### 3.10. Taxa Abundance of the Cecal Microbiome Communities

**Figure 8:**
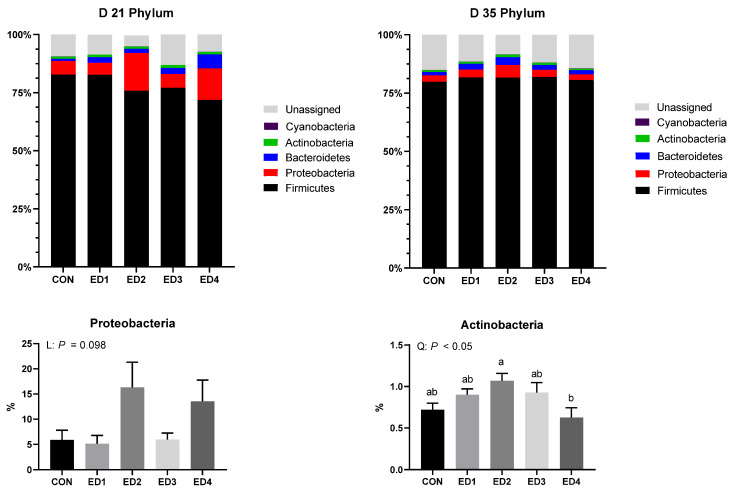
Phylum-level composition of the cecal microbial communities in the unchallenged control (CON); *Eimeria* dose 1 (ED1): *E. acervulina*: 31,250/*E. maxima*: 6250/*E. tenella*: 6250; *Eimeria* dose 2 (ED2): *E. acervulina*: 62,500/*E. maxima*: 12,500/*E. tenella*: 12,500; *Eimeria* dose 3 (ED3): *E. acervulina*: 125,000/*E. maxima*: 25,000/*E. tenella*: 25,000; and *Eimeria* dose 4 (ED4): *E. acervulina*: 250,000/*E. maxima*: 50,000/*E. tenella*: 50,000 groups. Oral gavage of *Eimeria* spp. Inoculum (1 mL of PBS) was performed on D 15. The phylum with statistical differences were presented. Experimental groups were compared using PROC MIXED followed by the Tukey’s HSD (honestly significant difference) test, and different letters mean significant differences (*p* < 0.05) among the experimental groups. Orthogonal polynomial contrasts were conducted to see linear and quadratic patterns among the experimental groups.

**Figure 9:**
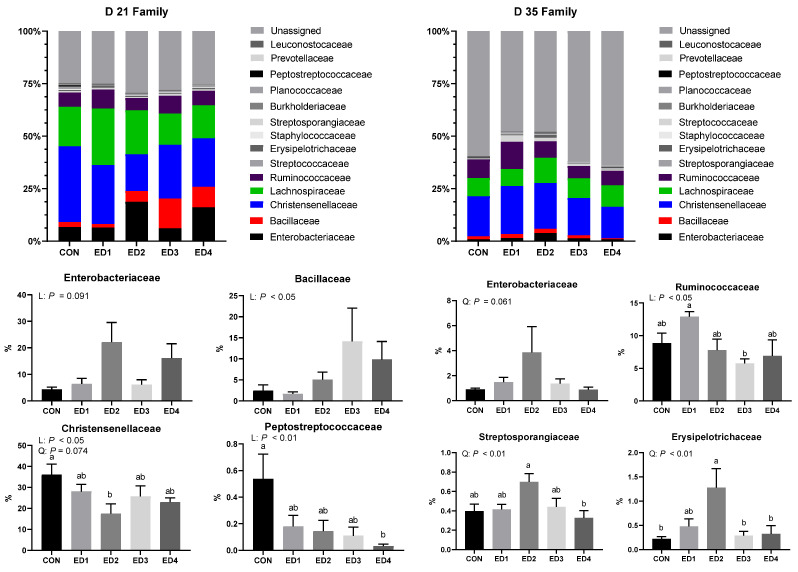
Family-level composition of the cecal microbial communities in the unchallenged control (CON); *Eimeria* dose 1 (ED1): *E. acervulina*: 31,250/*E. maxima*: 6250/*E. tenella*: 6250; *Eimeria* dose 2 (ED2): *E. acervulina*: 62,500/*E. maxima*: 12,500/*E. tenella*: 12,500; *Eimeria* dose 3 (ED3): *E. acervulina*: 125,000/*E. maxima*: 25,000/*E. tenella*: 25,000; and *Eimeria* dose 4 (ED4): *E. acervulina*: 250,000/*E. maxima*: 50,000/*E. tenella*: 50,000 groups. Oral gavage of *Eimeria* spp. inoculum (1 mL of PBS) was performed on D 15. The family with statistical differences were presented. Experimental groups were compared using PROC MIXED followed by the Tukey’s HSD (honestly significant difference) test, and different letters mean significant differences (*p* < 0.05) among the experimental groups. Orthogonal polynomial contrasts were conducted to see linear and quadratic patterns among the experimental groups.

As shown in Figure 8, increased *Eimeria* inoculation doses tended to increase the relative abundance of the phylum Proteobacteria (*p* = 0.098). Increased *Eimeria* inoculation doses tended to linearly enlarge the relative abundance of the family Enterobacteriaceae (Figure 9; *p* = 0.091). The relative abundance of the family Bacillaceae was enlarged in a linear trend by increased *Eimeria* inoculation doses (*p* < 0.05). Increased *Eimeria* inoculation doses linearly (*p* < 0.05) and quadratically (tendency; *p* = 0.074) reduced the relative abundance of the family Christensenellaceae, and the ED2 group had significantly lower the relative abundance of the family Christensenellaceae compared to the CON group. Increased *Eimeria* inoculation doses linearly reduced the relative abundance of the family Peptostreptococcaceae, and the ED4 group had a significantly lower relative abundance of the family Peptostreptococcaceae compared to the CON group.

On D 35, the relative abundance of the phylum Actinobacteria were quadratically enlarged by increased *Eimeria* inoculation doses (*p* < 0.05), and the ED4 group had a significantly lower relative abundance of the phylum Actinobacteria compared to the ED2 group (Figure 8). Increased *Eimeria* inoculation doses tended to increase the relative abundance of the family Enterobacteriaceae with a linear trend (*p* = 0.061; Figure 9). The relative abundance of the family Ruminococcaceae was linearly reduced by *Eimeria* infection (*p* < 0.05), and the ED3 group had a significantly lower relative abundance of the family Ruminococcaceae compared to the ED1 group. Increased *Eimeria* inoculation doses quadratically increased the relative abundance of the family Streptosporangiceae (*p* < 0.01), and the ED2 group had significantly higher relative abundance of the family Streptosporangiceae compared to the ED4 group. Increased *Eimeria* inoculation doses quadratically increased the relative abundance of the family Erysipelotrichaceae (*p* < 0.01), and the ED2 group had significantly higher relative abundance of the family Streptosporangiceae compared to the CON, ED3, and ED4 groups.

### 3.11. Eimeria Gene Expression

As shown in Table 12, different *Eimeria* doses did not modulate mRNA expression of *E. acervulina* and *E. tenella* genes including *APN*, flagella-related protein, elongation factors (*EF*), and gametocyte proteins (*GAM*) in broilers on D 21 (*p* > 0.1). However, higher *Eimeria* inoculation doses linearly upregulated the gene expression of *E. maxima APN* (*p* < 0.05), and the ED3 group had significantly higher *E. maxima APN* compared to the ED1 and ED2 groups. Higher *E. maxima* doses linearly increased the gene expression of *E. maxima* EF2 (*p* < 0.05). Higher *E. maxima* doses linearly upregulated the gene expression of *E. maxima GAM56* (*p* < 0.01), and the ED3 group had significantly higher gene expression of *E. maxima GAM56* compared to the ED1 and ED2 groups. Gene expression of *E. maxima GAM82* was linearly increased by higher doses of *Eimeria* (*p* < 0.01), and the ED3 group had a significantly higher gene expression of *E. maxima GAM82* compared to the ED1 group.

### 3.12. Body Composition

The body composition of broilers infected with *Eimeria* spp. on D 21, 28, and 35 is shown in Table 13. On D 21, total weight was linearly reduced by increased *Eimeria* inoculation doses (*p* < 0.05), and the ED4 group had a significantly lower total weight compared to the CON group. Increased *Eimeria* infection doses linearly reduced fat weight (*p* < 0.05) and lean weight (*p* < 0.01). No differences were observed on D 28 (*p* > 0.1). On D 35, fat weight was linearly reduced by increased *Eimeria* inoculation doses (*p* < 0.05), and the ED4 group tended to have lower fat weight compared to the CON group (*p* = 0.055). The lean weight tended to be linearly decreased by increased *Eimeria* inoculation doses (*p* = 0.068). Fat percentage tended to be reduced with a linear trend by increased *Eimeria* inoculation doses (*p* = 0.060), and lean percentage tended to be increased with a linear trend by increased *Eimeria* inoculation doses (*p* = 0.059). Lean:fat ratio was linearly reduced by increased *Eimeria* inoculation doses (*p* < 0.05).

## 4. Discussion

The purpose of the study was to investigate the effects of different *Eimeria* inoculation doses on growth performance, litter moisture content, nutrient digestion and absorption, incidence and severity of FPD, gut microbiota, oocyst shedding, *Eimeria* gene expression, and body composition in broilers raised in floor pens for 35 days. Cross contamination among pens in the *Eimeria* infection studies in floor pens would be problematic [27]. To minimize cross contamination between the unchallenged group and *Eimeria* challenged groups, an anti-coccidial drug (Coban 90) was supplemented in the unchallenged group (CON group) in the current study. The dose of Coban 90 (500 mg/kg) was determined based on our previous study (unpublished). Broilers challenged with *Eimeria* spp. and fed 500 mg/kg of Coban 90 showed improved body weight compared to the challenged group without Coban 90, and their BW was similar to that of the non-challenged group. Furthermore, the supplementation of 500 mg/kg of Coban 90 did not adversely affect the growth performance of broiler chickens without an *Eimeria* challenge. However, although workers were extra careful during the entire experimental period, there was still *Eimeria* cross contamination (e.g., oocyst shedding and lesion score) to the unchallenged group in the current study. These results indicate that supplementation of Coban 90 at 500 mg/kg did not completely inhibit the colonization of *Eimeria* spp. in broilers even with indirect infection [28]. However, significant statistical differences in body weight (BW) were observed between the control group and the *Eimeria* challenged groups on Day 35. Additionally, throughout the current study, the control group consistently had lesion scores for *Eimeria* spp. lower than 0.5 and a minimum gut permeability. These findings suggest that the level of Eimeria infection in the CON group did not have a significant impact on serving as the negative control for the CON group. The DFI was measured during the entire experiment period to check the severity of *Eimeria* infection and whether *Eimeria* re-infection occurred in floor pen conditions. The DFI could be one of the powerful and non-invasive parameters to indicate incidence and severity of *Eimeria* infection in broilers. Measuring DFI has benefits in easiness and time over other non-invasive methods including measuring core body temperature or fecal moisture content. Our previous study [29] showed that *Eimeria* spp. Infection reduced ADFI in broilers in the acute phase (0 to 6 days post infection (dpi)). The time points 5 dpi and 6 dpi were considered as the peak time points for *Eimeria* infection according to our previous studies based on the results of gut permeability and daily feed intake [14,29]. In the current study, DFI was dramatically decreased on 5 and 6 dpi and continued to be linearly reduced by increased *Eimeria* inoculation doses from 11 to 14 dpi (D 26 to 29), which potentially indicates that re-infection of *Eimeria* has occurred in the current study. This was supported by the results of *Eimeria* lesion and oocyst shedding of *Eimeria* spp. on D 28 and 35, while the average values for *Eimeria* lesion scores on D 35 were below 1 out of 4 in the current study. Once exposed to *Eimeria* infection, chickens develop strong humoral and cellular immunity against re-infection of *Eimeria* [30], which may demonstrate no statistical differences in gut permeability and intestinal morphology in broilers after the acute phase in the current study. Still, our current study showed that reinfection of *Eimeria* was able to decrease feed intake of broilers in the floor pen conditions. Reduced feed intake in broilers infected with *Eimeria* spp. might be due to alternation in immune response and endocrine system in broilers [31,32].

In the acute phase (0 to 6 dpi; D 15 to 21), reduced feed intake with impaired feed efficiency dramatically decreased BW and ADG in broilers infected with *Eimeria* spp. in the current study, which was in agreement with Teng et al. [14]. On D 21 to 28, BW and ADG were reduced, along with decreased ADFI, without affecting feed efficiency in *Eimeria*-infected broilers. These results indicate that severe *Eimeria* infection reduced growth rate of broilers by damaging the capacity of nutrient digestion and absorption and reducing the feed intake in broilers, but re-infection of *Eimeria* decreased growth rate via decreasing only feed intake in broilers. These indicate that decreasing feed intake could be a sensitive sign in *Eimeria*-infected broilers. On D 35, *Eimeria* infection reduced only BW without affecting ADG, ADFI, and FCR in the *Eimeria* challenged groups compared to the CON group on D 35. These results were in consistent with a study reported that *Eimeria* inoculation on D 15 decreased BW in broilers on D 42 in floor pens [33]. In previous studies, compensatory growth happened in broilers infected with *E. maxima* in the recovery phase (6 to 13 dpi) [12] and in broilers challenged with *E. acervulina* and *E. maxima* (14 to 21 dpi) [34] by improving feed intake or feed efficiency after the acute phase of *Eimeria* infection. However, potentially, infection of *E. acervulina*, *E. maxima*, and *E. tenella* would not induce compensatory growth in the current study because they induced severe damage in the gastrointestinal tract of broilers. More studies are required to explain the potential mode of actions of *Eimeria* infection on reduced feed intake and to elucidate compensatory growth after *Eimeria* infection in broilers.

Higher *Eimeria* inoculation doses above the threshold where the maximal reproductive potential reached may result in reduced oocyst shedding and downregulated *Eimeria* genes relating to viability and sexual reproduction due to crowding effects in the gastrointestinal tract [35]. In our previous study, while higher doses of *E. tenella* increased oocyst shedding 5 to 6 dpi, oocyst shedding 6 to 8 dpi was not affected by challenging doses [36]. This potentially designates that *Eimeria* can control themselves to determine maximal oocyst production in broilers, which is called crowding effect [35,37]. In the current study, the ED4 group (the highest dose group) had numerically similar oocyst shedding of E. maxima and *E. tenella* compared to the ED2 and ED3 groups in the cloaca content on D 22. *Eimeria* oocysts were counted in the cloaca content and litter instead of feces because it is not feasible to collect fresh fecal samples in the litter condition. To elucidate crowding effects of *Eimeria* spp., whole duodenal, jejunal, and cecal tissue samples were collected on 6 dpi, one day prior to peak date (7 dpi) for oocyst production of *E. maxima* and *E. tenella* [38], and gene expression of *APN*, *EF2*, *GAM56*, and *GAM82* of *E. maxima* were modulated by increased *Eimeria* inoculation doses along with quadratically modulated oocyst production of *E. maxima* in the current study. The *APN*, *EF*, and *GAM* of *Eimeria* spp. play important roles in *Eimeria* viability and sexual reproduction to produce oocysts [24,39,40]. The inconsistency between linearly increased gene expression of *APN*, *EF*, and *GAM* and quadratically modulated oocyst shedding of *E. maxima* is still in question because only transcriptional level was analyzed in the current study. However, our current study showed that different inoculation doses of *Eimeria* can alter *Eimeria* gene expression. Increased inoculation doses only affected *E. maxima* mRNA expression in the current study potentially because *E. maxima* is more sensitive to crowding effects due to their largest size among chicken *Eimeria* spp. [41].

In the current study, ileal digesta and litter moisture contents were increased due to *Eimeria* infection in the acute phase. In our previous study, *E. tenella* infection did not increase ileal digesta moisture content on 5 to 7 dpi and even decreased ileal moisture content on 6 dpi [36]. Increased ileal moisture content is mainly due to infection of *E. acervulina* and *E. maxima* in broilers. Increased digesta moisture content may imply shortened digesta transit time, which can reduce the capacity of nutrient digestion and absorption [42] and is associated with reduced nutrient digestibility in the current study. Litter moisture content was linearly increased in the acute phase and was quadratically increased by *Eimeria* infection on D 35 in the current study. Many studies demonstrated that litter moisture is closely associated with FPD in broiler chickens [6,43,44]. FPD is a type of skin inflammation that induces necrotic lesions on the plantar surface of foot pad in broilers [45]. FPD can reduce the marketability of chicken feet, be an entry route for pathogenic bacteria, cause lameness, and reduce the growth performance of broiler chickens [46]. Increased litter moisture content due to *Eimeria* infection can increase the incidence and severity of FPD in broilers by increasing the litter ammonia concentration [47,48]. The litter moisture content on D 35 and severity/incidence of FPD on D 35 showed similar trends among the experimental groups in the current study, while the severity and incidence of FPD was mild potentially because of the dry conditions of the room and early slaughter age (D 35) in the current study. A previous study by El-Wahab et al. [43] showed that litter moisture should be above 35% to induce FPD in poultry. *Eimeria* infection still has the potential to increase the incidence and severity of FPD by increasing litter moisture content in broilers.

Gut permeability measured by FITC-D4 is an important indicator to represent functionality and integrity of gut in broilers [49]. Increased gut permeability indicates that more pathogens and toxins can permeate into the blood stream across the epithelial layer, which can cause systemic infection in broilers [50]. In the current study, *Eimeria* infection increased gut permeability on 5 dpi in broilers, which is consistent with our previous study [14]. Our previous study demonstrated that *E. maxima* infection had a significant impact on increasing gut permeability [51], whereas *E. tenella* infection did not affect gut permeability in broilers [36]. Potentially, the infection of *E. acervulina* and *E. maxima* disrupts tight junction proteins and the mucus layer, which play an important role in maintaining gut barrier integrity, and this would make the intestinal wall thinner (more permeable) in broilers [52,53]. In contrast, Vicuña et al. [49] showed that FITC-D4 can be deposited in the cecal tissue, and *E. tenella* infection did not increase gut permeability potentially because *E. tenella* infection thickened the intestinal wall in the ceca [36]. No differences were observed in the gut permeability on D 27 in the current study, and this suggests that the re-infection of *Eimeria* did not severely damage gut functionality and integrity, while it reduced feed intake in broilers in the floor pen conditions.

Reduced growth performance and feed efficiency might be mainly attributed to the reduced capacity of nutrient digestion and absorption in *Eimeria*-infected broilers. In the current study, the AID of CP was linearly reduced by *Eimeria* infection in broilers in the acute phase (0 to 6 dpi), which is consistent with our previous study by Teng et al. [14], which reported that *Eimeria* infection reduced the AID of CP in *Eimeria*-infected broilers. *E. acervulina* and *E. maxima* are the main *Eimeria* spp. that directly reduce the AID of CP because they inhabit in the duodenum and jejunum, respectively [54,55]. Our previous study by Choi et al. [36] reported that *E. tenella* infection in the ceca did not directly affect AID in the acute (6 dpi) and recovery phase (9 dpi) in broilers. The AID method does not account for the endogenous loss of nutrients in the gastrointestinal tract of broilers [54]. Both nutrient disappearance and increased endogenous loss may have affected the AID of CP values in the *Eimeria*-infected broilers because *Eimeria* infection impaired the intestinal morphology and activities of jejunal brush border digestive enzymes in the current study. *Eimeria* infection is known to increase endogenous losses of proteins (e.g., plasma proteins, mucin, and cell debris) in the gastrointestinal tract during *Eimeria* colonization and reproduction activities [56,57]. The AID of CF was dramatically reduced by *Eimeria* infection, and AID values of CF were negative in the ED2, ED3, and ED4 groups in the current study. Consistently, Ghareeb et al. [58] reported that *E. maxima* infection dramatically reduced the AID of CF in broilers. The negative values for AID of CF indicate that there were high endogenous losses in *Eimeria*-infected broilers. Endogenous fat from the gastrointestinal tract includes bile, cell debris, intestinal secretions, and microbial lipids [59]. Adams et al. [60] demonstrated that infection of *E. acervulina* decreased the secretion of bile, which has an essential role in fat digestion by emulsifying fat in the gastrointestinal tract. Potentially, reduced bile secretion and increased cell debris from the gastrointestinal tract may have decreased fat digestibility and increased endogenous loss of fat, which resulted in negative values for the AID of CP in *Eimeria*-infected broilers in the current study. However, more studies are needed to specify the factors such as bile, cell debris, intestinal secretions, and microbial lipids that increased endogenous losses of fat in *Eimeria*-infected broilers.

Intestinal morphology is an important indicator to represent capacity of nutrient digestion and absorption in the gastrointestinal tract of chickens [61]. In the current study, infection of *E. acervulina* and *E. maxima* reduced VH and increased CD in both duodenum and jejunum on D 21. These results agree with several previous studies [62,63]. The reduced VH and increased CD implies there was high tissue turnover in the intestine by *Eimeria* infection because bigger crypts, which are reservoirs for enterocytes, indicates a high demand for new tissue in the villus [62,64]. Impaired jejunal morphology is highly associated with reduced activities of jejunal maltase in the current study. This is because mature enterocytes in the villus, which mainly express brush border digestive enzymes, would be quickly deceased due to a higher turnover rate in *Eimeria* infection conditions [65]. Furthermore, negatively modulated duodenal and jejunal morphology would explain decreased AID of CP and EE in the current study. While no differences were observed in the jejunal morphology on D 35, duodenal VH:CD were quadratically reduced with increased duodenal CD in *Eimeria*-infected broilers in the current study. Reduced VH:CD due to increased CD indicates more energy and nutrients are required for gut maintenance, which can result in growth retardation in broilers [62]. However, while statistical differences were not observed, the ED3 and ED4 groups exhibited numerically higher duodenal VH and numerically lower VH:CD compared to the control group on D 35. This numerical trend suggests that gut maintenance and new tissue generation may have occurred on D 35. This result also indicates that negative effects of *E. acervulina* infection lasted through to D 35 in broilers in the current study.

Chicken ceca have only crypts without villus, similarly to the colon of mammals (humans and pigs) [66]. In the current study, *E. tenella* infection deepened the cecal crypts as we observed in our previous study [36]. However, it is still uncertain whether thickened cecal wall due to *E. tenella* infection decreases the permeation of microbial metabolites (e.g., endotoxins and short chain fatty acids) across epithelium. In the current study, *Eimeria* infection negatively affected cecal microbiota in the acute and chronic phases. In the current study, *Eimeria* infection increased the phylum Proteobacteria and the family Enterobacteriaceae in the acute phase, which includes diverse pathogenic bacteria such as *Escherichia coli*, *Salmonella* spp., *Vibrio* spp., and *Pseudomonas* spp. [67]. Moreover, in the acute phase, *Eimeria* infection reduced the relative abundance of the family Christensenellaceae and Peptostreptococcaceae, which play important roles in fiber degradation and short chain fatty acid production in broilers [68]. Potentially, negatively altered cecal microbiota may have reduced volatile fatty acids (VFA) production in the ceca, and this would explain the reduced activities of SAP, which needs VFA production [36,69]. Whereas alpha diversity indices in the cecal microbial communities on D 21 were not affected by *Eimeria* infection, all alpha diversity indices including pielou evenness (evenness), faith phylogenetic diversity (biodiversity based on phylogeny), shannon entropy (richness and evenness), and observed features (richness) were linearly decreased on D 35 in the current study. Reduced alpha diversity indices suggest the presence of unhealthier and immature gut microbiota in chickens [70]. Furthermore, the relative abundance of the family Enterobacteriaceae was quadratically increased by *Eimeria* infection, and the relative abundance of the family Ruminococcaceae, which play an important role in fiber degradation and short-chain fatty acid production in the ceca of chickens [71], was linearly reduced by *Eimeria* infection on D 35 in the current study. These results imply that *Eimeria* infection on D 15 still negatively influenced cecal microbiota in broilers on D 35. Our previous study showed that *E. tenella* infection negatively influenced cecal microbiota toward increasing the abundance of pathogenic bacteria and reducing microbial VFA (e.g., an important energy source for chickens) production mainly by impairing the mucosal immune system of broilers and increasing the protein concentration in the cecal content [72]. While *E. tenella* infection would be the main factor to alter cecal microbiota, the infection of *E. acervulina* and *E. maxima* potentially can affect cecal microbiota by increasing endogenous loss of proteins and undigested dietary proteins in the gastrointestinal tract because the phylum Proteobacteria mainly ferment protein sources for their growth and reproduction [73,74,75]. More studies are required to investigate whether the infection of *E. acervulina* and *E. maxima* itself can alter cecal microbiota in broilers.

Body composition is a crucial parameter in broiler production because body composition is closely associated with meat yield and quality [76]. In the current study, body composition of broilers was altered by *Eimeria* infection. On D 35, the lean:fat ratio was linearly enhanced, and fat accumulation was reduced by increased *Eimeria* infection doses in the current study. Potentially, *Eimeria* infection stimulated the immune system, resulting in the excessive usage of energy sources (e.g., fat and glycogen) in broilers [77,78]. Furthermore, reduced fat digestibility and decreased cecal VFA production due to negatively altered microbiota on D 21 could result in decreased fat accumulation as a chronic effect on D 35. These results suggest that *Eimeria* infection can influence the body composition and meat quality of broilers.

## 5. Conclusions

In conclusion, *Eimeria* infection negatively affected the growth performance, gut health, gut barrier integrity, nutrient digestion and absorption, gut microbiota, and body composition of broilers in the acute phase. Increased *Eimeria* inoculation doses modulated the relative mRNA expression of *Eimeria* genes relating to viability and sexual reproduction. *Eimeria* infection negatively affected the growth performance, gut microbiota, FPD, and body composition in broilers, and the negative effects were prolonged to D 35 in the floor pen conditions.

## Figures and Tables

**Table 1:** Ingredients and nutrient compositions of diets (as-fed basis).

Items	D 15 to 28	D 28 to 35
Ingredients (kg/ton)		
Corn	675.26	693.66
Soybean meal (480 g crude protein/kg)	262.75	238.77
Soybean oil	17.70	23.05
Dicalcium phosphate	12.62	12.81
Limestone	10.58	10.66
Sand ^1^	7	7
Titanium dioxide ^2^	3	3
Common Salt	3.45	3.47
DL-Methionine 99%	2.65	2.48
L-Lysine HCl 78%	2.01	2.15
Vitamin Premix ^3^	1	1
Mineral Premix ^4^	0.8	0.80
Choline 60%	0.70	0.60
L-threonine	0.48	0.54
Total	1000	1000
Calculated energy and nutrient value, %		
Metabolizable energy, kcal/kg	3100	3150
Crude fat	4.39	4.972
Crude protein	18	17
SID ^5^ methionine	0.544	0.515
SID total sulfur amino acids	0.800	0.760
SID lysine	1.02	0.970
SID threonine	0.660	0.630
Total calcium	0.760	0.760
Available phosphate	0.380	0.380

^1^ The diet for the CON group contained 0.05% anticoccidial drug (Coban 90, Elanco Animal Health, Greenfield, IN, USA) in the sand part. ^2^ Titanium dioxide 3 g/kg (Acros Organics, Morris Plains, NJ, USA). ^3^ Vitamin mix included the following per kg: vitamin A (IU): 3,527,160; vitamin D3 (ICU): 1,399,921; Vitamin E (IU): 19,400; Vitamin B12 (mg): 8.8; Menadione (mg): 1102; Riboflavin (mg): 3527; d-Pantothenic acid (mg): 5467; Thiamin (mg): 970, Niacin (mg): 20,282; Vitamin B6 (mg): 1455; Folic acid (mg): 573; and Biotin (mg): 79. ^4^ Mineral mix included the following per kg: Ca (g): 0.72; Mn (g): 3.04; Zn (g): 2.43; Mg (g): 0.61; Fe (g): 0.59; Cu (g): 22.68; I (g): 22.68; Se (g): 9.07. ^5^ SID: standard ileal digestible amino acid.

**Table 2:** Footpad dermatitis (FPD) scoring system according to Sorin et al. [17].

FPD Score	Description
0	No lesion
1	FPD covers less than 50% of the food pad
2	FPD covers more than 50% of the food pad

**Table 3:** Primers used in the study.

Genes	Sequence, 5′ to 3′	Amplicon	Accession Number
Beta actin	F: CAACACAGTGCTGTCTGGTGGTA	205	NM_205518.2
	R: ATCGTACTCCTGCTTGCTGATCC		
*GAPDH* ^1^	F: GCTAAGGCTGTGGGGAAAGT	161	NM_204305.2
	R: TCAGCAGCAGCCTTCACTAC		
*Eimeria acervulina*			
*18s*	F: CTGCGAATGGCTCATTAAAA	123	KT184333.1
	R: AATAAACACAGCCCCTCCAG		
*APN* ^2^	F: CGTCTCCTCATTGATGCTGT	109	XM_013396512.1
	R: GCCTTCTGTCTCCTTTCAGG		
Flagella-related protein	F: AGTGTTTAGCCCATCGAACC	123	XM_013394342.1
	R: TTGCCACATTAACGACAGGT		
*EF1* ^3^	F: CCCAACAGCTCTGAGACAAA	118	XM_013397273.1
	R: TGTATAGGCCGAAAGCAATG		
*EF2*	F: GATTCCGTTGATGGTGTTTG	107	XM_013392455.1
	R: AGAAGTGCGCGATCTACCTT		
*GAM56* ^4^	F: ATCAGCAGCAGTCCTACACG	103	MK519446.1
	R: GGTGTGCATAGCCATAGGTG		
*GAM82*	F: GGCAGCACTTGAAGCAATAA	117	MK519447.1
	R: CGACCGACAATGTTTCTGAC		
*Eimeria maxima*			
*18s*	F: TCGCGTCTCTAATGATCGTC	110	AF027724.1
	R: TCTGCAATTCACAATGCGTA		
*APN*	F: TTTCGCCGTTGATTCTGTAG	124	XM_013483142.1
	R: CTCCCCATTCAAGACCAAGT		
Flagella-related protein	F: GAGTTCCAGTCGTGGGATTT	116	XM_013478390.1
	R: ACTGCCTGAAGCAGAAAGGT		
*EF1A*	F: TTGATGATAGCAGCGTCTCC	124	GO305089.1
	R: GATGCCCTACAGCACACATT		
*EF2*	F: GATGGAAAGGGAGAACAGGA	125	GO305837
	R: ACAGAATCCACGACGACAAG		
*GAM56*	F: CTTCCCTGAAACCCCTATGA	121	AY129951.2
	R: TGAGGCTACGAAATGTGAGC		
*GAM82*	F: AGGTACCCCAGCTATGATGC	109	AY179510.2
	R: CACGCGAGTATATGCTGGAT		
*Eimeria tenella*			
*18s*	F: GTGCAAGGTTACGGAAGGAT	113	XM_013373467.1
	R: CTACTGCTGTGTGGGTTGCT		
*APN*	F: TTCAAGACAGTTTGCCGAAG	119	ETH_00013105
	R: GCACAACCTCTGCACCTTTA		
Flagella-related protein	F: GAGACAGGGCATTTGCTTC	120	XM_013378497.1
	R: TGGTAGAAGCCGTAGGCAAT		
*EF1A*	F: TGATCGTGGGGATAAACAAA	115	CD665486.1
	R: GGGTTGTAGCCCACTGTCTT		
*GAM22*	F: TAGCCACCCTAGTCGGTTTC	100	MH445412.1
	R: ATCGCTTCTGGATCGATTTC		
*GAM56*	F: AGATGGGCACTTACCAGGAG	112	XM_013376832.1
	R: AAGTTCTCCAGCCACTGGTC		

^1^ *GAPDH*: glyceraldehyde 3-phosphate dehydrogenase. ^2^ *APN*: aminopeptidase N. ^3^ *EF*: elongation factor. ^4^ *GAM*: gametocyte protein.

**Table 4:** Effects of different *Eimeria* infection doses on growth performance parameters including body weight (BW; g), average daily gain (ADG; g/d), average daily feed intake (ADFI; g/d), and feed conversion ratio (FCR, g/g) in broilers ^1^.

	CON	ED1	ED2	ED3	ED4	SEM ^2^	*p* Value	Linear	Quadratic
Initial BW	440.8	442.4	443.0	444.3	442.8	18.9	0.998	0.809	0.845
D 15 to 21									
BW	859.76 ^a^	769.97 ^b^	747.12 ^b^	719.24 ^bc^	689.27 ^c^	30.13	<0.001	<0.001	0.020
ADG	69.83 ^a^	54.60 ^b^	50.68 ^bc^	45.82 ^cd^	41.08 ^d^	2.88	<0.001	<0.001	<0.001
ADFI	99.99 ^a^	88.44 ^b^	87.30 ^bc^	82.44 ^cd^	80.63 ^d^	3.51	<0.001	<0.001	0.007
FCR	1.43 ^d^	1.62 ^c^	1.72 ^bc^	1.80 ^b^	1.97 ^a^	0.080	<0.001	<0.001	0.598
D 21 to 28									
BW	1478.8 ^a^	1312.0 ^b^	1301.3 b	1241.7 ^b^	1230.6 ^b^	51.37	<0.001	<0.001	0.003
ADG	88.43 ^a^	77.43 ^b^	79.17 ^b^	74.63 ^b^	77.33 ^b^	4.98	<0.001	<0.001	0.01
ADFI	145.83 ^a^	134.45 ^b^	133.06 ^b^	126.48 ^b^	129.52 ^b^	6.23	<0.001	<0.001	0.02
FCR	1.65	1.74	1.68	1.70	1.68	0.060	0.211	0.847	0.136
D 28 to 35									
BW	2237.9 ^a^	2049.5 ^b^	2057.6 ^b^	1975.8 ^b^	1961.7 ^b^	64.59	<0.001	<0.001	0.015
ADG	108.44	105.37	108.04	104.87	104.45	5.02	0.519	0.202	0.946
ADFI	180.15	181.55	182.48	180.80	180.50	4.48	0.902	0.992	0.389
FCR	1.66	1.73	1.69	1.73	1.73	0.06	0.314	0.110	0.599
Whole phase									
ADG	89.86 ^a^	80.36 ^b^	80.73 ^b^	76.57 ^b^	75.95 ^b^	2.92	<0.001	<0.001	0.006
ADFI	144.09 ^a^	137.13 ^b^	136.63 ^b^	132.28 ^b^	132.70 ^b^	3.85	<0.001	<0.001	0.076
FCR	1.59 ^c^	1.70 ^b^	1.70 ^b^	1.74 ^ab^	1.78 ^a^	0.04	<0.001	<0.001	0.146

^1^ CON (unchallenged control); ED1 (*Eimeria* dose 1): *E. acervulina*: 31,250/*E. maxima*: 6250/*E. tenella*: 6250; ED2 (*Eimeria* dose 2): *E. acervulina*: 62,500/*E. maxima*: 12,500/*E. tenella*: 12,500; ED3 (*Eimeria* dose 3): *E. acervulina*: 125,000/*E. maxima*: 25,000/*E. tenella*: 25,000; ED4 (*Eimeria* dose 4): *E. acervulina*: 250,000/*E. maxima*: 50,000/*E. tenella*: 50,000. Oral gavage of *Eimeria* spp. was performed on D 15. Experimental groups were compared using PROC MIXED followed by the Tukey’s HSD (honestly significant difference) test. Different letters in the same row means significant differences (*p* < 0.05) among the experimental groups. Orthogonal polynomial contrasts were conducted to see linear and quadratic patterns among the experimental groups. ^2^ Standard errors of the means.

**Table 5:** Effects of different *Eimeria* infection doses on gut permeability concentration (mg/mL) of fluorescein isothiocyanate–dextran (molecular weight: 4 kDa; FITC-D4) in broilers on D 20 and D 27 ^1^.

	CON	ED1	ED2	ED3	ED4	SEM ^2^	*p* Value	Linear	Quadratic
D 20	0.002 ^c^	0.057 ^c^	0.125 ^bc^	0.355 ^a^	0.326 ^ab^	0.125	<0.001	<0.001	0.976
D 27	0.195	0.551	0.530	0.387	0.462	0.3	0.286	0.361	0.157

^1^ CON (unchallenged control); ED1 (*Eimeria* dose 1): *E. acervulina*: 31,250/*E. maxima*: 6250/*E. tenella*: 6250; ED2 (*Eimeria* dose 2): *E. acervulina*: 62,500/*E. maxima*: 12,500/*E. tenella*: 12,500; ED3 (*Eimeria* dose 3): *E. acervulina*: 125,000/*E. maxima*: 25,000/*E. tenella*: 25,000; ED4 (*Eimeria* dose 4): *E. acervulina*: 250,000/*E. maxima*: 50,000/*E. tenella*: 50,000. Oral gavage of *Eimeria* spp. was performed on D 15. Experimental groups were compared using PROC MIXED followed by the Tukey’s HSD (honestly significant difference) test. Different letters in the same row means significant differences (*p* < 0.05) among the experimental groups. Orthogonal polynomial contrasts were conducted to see linear and quadratic patterns among the experimental groups. ^2^ Standard errors of the means.

**Table 6:** Effects of different *Eimeria* infection doses on ileal digesta and litter moisture content (%) in broilers ^1^.

	CON	ED1	ED2	ED3	ED4	SEM ^2^	*p* Value	Linear	Quadratic
Ileum digesta									
D 21	81.3 ^b^	85.1 ^ab^	86.3 ^ab^	86.6 ^ab^	89.9 ^a^	3.04	0.002	<0.001	0.697
D 35	86.3 ^b^	89.5 ^ab^	87.9 ^ab^	90.2 ^ab^	93.0 ^a^	3.62	0.040	0.005	0.545
Litter									
D 22	26.8	32.8	30.0	33.0	37.0	9.16	0.415	0.098	0.891
D 28	20.1	25.6	17.9	22.5	22.7	6.82	0.383	0.816	0.873
D 35	24.1	28.4	25.8	25.0	23.0	3.35	0.100	0.200	0.047

^1^ CON (unchallenged control); ED1 (*Eimeria* dose 1): *E. acervulina*: 31,250/*E. maxima*: 6250/*E. tenella*: 6250; ED2 (*Eimeria* dose 2): *E. acervulina*: 62,500/*E. maxima*: 12,500/*E. tenella*: 12,500; ED3 (*Eimeria* dose 3): *E. acervulina*: 125,000/*E. maxima*: 25,000/*E. tenella*: 25,000; ED4 (*Eimeria* dose 4): *E. acervulina*: 250,000/*E. maxima*: 50,000/*E. tenella*: 50,000. Oral gavage of *Eimeria* spp. inoculum (1 mL of PBS) was performed on D 15. Experimental groups were compared using PROC MIXED followed by the Tukey’s HSD (honestly significant difference) test. Different letters in the same row means significant differences (*p* < 0.05) among the experimental groups. Orthogonal polynomial contrasts were conducted to see linear and quadratic patterns among the experimental groups. ^2^ Standard errors of the means.

**Table 7:** Effects of different doses of *Eimeria* spp. on oocyst shedding of *Eimeria acervulina*, *Eimeria maxima*, and *Eimeria tenella* in the cloaca content and litter of broilers on D 22, D 28, and D 35 ^1^.

	CON	ED1	ED2	ED3	ED4	SEM ^2^	*p* Value	Linear	Quadratic
D 22/Cloaca									
*E. acervulina*	0	161,677	619,889	716,744	1,497,462	1,003,493	0.12	0.011	0.583
*E. maxima*	0 ^b^	125,476 ^ab^	227,080 ^a^	100,460 ^ab^	129,101 ^ab^	99,216	0.018	0.081	0.012
*E. tenella*	0	80,162	407,110	203,800	466,214	329,393	0.101	0.02	0.751
D 22/Litter									
*E. acervulina*	0 ^b^	14,626 ^b^	38,062 ^ab^	86,810 ^a^	87,311 ^a^	34,489	<0.001	<0.001	0.956
*E. maxima*	0	27,237	25,777	23,110	12,657	16,497	0.0429	0.329	0.005
*E. tenella*	0 ^b^	1201 ^b^	25,437 ^a^	24,141 ^a^	18,550 ^ab^	13,424	0.004	0.002	0.068
D 28/Cloaca									
*E. acervulina*	1074 ^b^	185,269 ^a^	35,218 ^ab^	66,520 ^ab^	35,088 ^ab^	104,594	0.048	0.71	0.13
*E. maxima*	0	982.17	982.17	982.17	982.17	3449	0.041	0.12	0.018
*E. tenella*	0 ^b^	6111.7 ^ab^	11,395 ^a^	2804.88 ^ab^	2008.89 ^ab^	5956	0.025	0.927	0.005
D 28/Litter									
*E. acervulina*	2444	939	2681	2679	5044	3341	0.352	0.12	0.251
*E. maxima*	602	496	146	0	1001	932	0.39	0.804	0.102
*E. tenella*	1593	1753	1167	2922	1437	2253	0.706	0.771	0.785
D 35/Cloaca									
*E. acervulina*	31,641	11,185	14,544	27,598	1013	31,505	0.457	0.281	0.958
*E. maxima*	22,004	1909.96	0	552	7407	21,874	0.394	0.29	0.104
*E. tenella*	3745	37,915	992	1485	305	31,115	0.195	0.291	0.49
D 35/Litter									
*E. acervulina*	26,572	22,367	15,392	10,743	19,730	30,159	0.908	0.522	0.539
*E. maxima*	6500	0	1409	3480	2347	5556	0.347	0.507	0.191
*E. tenella*	2524.98	14,995	2117.5	8523.45	7920.53	14,321	0.531	0.817	0.756

^1^ CON (unchallenged control); ED1 (*Eimeria* dose 1): *E. acervulina*: 31,250/*E. maxima*: 6250/*E. tenella*: 6250; ED2 (*Eimeria* dose 2): *E. acervulina*: 62,500/*E. maxima*: 12,500/*E. tenella*: 12,500; ED3 (*Eimeria* dose 3): *E. acervulina*: 125,000/*E. maxima*: 25,000/*E. tenella*: 25,000; ED4 (*Eimeria* dose 4): *E. acervulina*: 250,000/*E. maxima*: 50,000/*E. tenella*: 50,000. Oral gavage of *Eimeria* spp. inoculum (1 mL of PBS) was performed on D 15. Experimental groups were compared using PROC MIXED followed by the Tukey’s HSD (honestly significant difference) test. Different letters in the same row means significant differences (*p* < 0.05) among the experimental groups. Orthogonal polynomial contrasts were conducted to see linear and quadratic patterns among the experimental groups. ^2^ Standard errors of the means.

**Table 8:** Effects of different *Eimeria* infection doses on apparent ileal digestibility (%) of dry matter (DM), organic matter (OM), ash, crude protein (CP), and crude fat (CF) in broilers on D 21 and D 35 ^1^.

	CON	ED1	ED2	ED3	ED4	SEM ^2^	*p* Value	Linear	Quadratic
D 21									
DM	52.49	56.45	48.09	47.06	47.14	13.99	0.726	0.295	0.985
OM	55.16	58.38	50.38	49.79	49.35	13.29	0.716	0.268	0.996
Ash	7.87	24.24	9.76	1.42	10.17	28.19	0.72	0.64	0.84
CP	67.92 ^a^	62.04 ^ab^	53.82 ^ab^	53.15 ^ab^	46.50 ^b^	11.3	0.037	0.002	0.738
CF	53.47 ^a^	11.22 ^ab^	−8.54 ^b^	−9.23 ^b^	−11.21 ^b^	31.34	0.013	0.002	0.056
D 35									
DM	68.25	71.46	67.24	69.68	68.25	2.65	0.358	0.761	0.687
OM	69.20	72.74	68.73	71.38	70.53	7.75	0.398	0.83	0.751
Ash	46.56	45.21	36.75	36.40	25.61	6.57	0.045	0.005	0.504
CP	76.20	78.40	75.72	77.16	76.98	2.71	0.764	0.957	0.923
CF	45.90	58.08	54.81	56.71	48.69	15.12	0.875	0.899	0.347

^1^ CON (unchallenged control); ED1 (*Eimeria* dose 1): *E. acervulina*: 31,250/*E. maxima*: 6250/*E. tenella*: 6250; ED2 (*Eimeria* dose 2): *E. acervulina*: 62,500/*E. maxima*: 12,500/*E. tenella*: 12,500; ED3 (*Eimeria* dose 3): *E. acervulina*: 125,000/*E. maxima*: 25,000/*E. tenella*: 25,000; ED4 (*Eimeria* dose 4): *E. acervulina*: 250,000/*E. maxima*: 50,000/*E. tenella*: 50,000. Oral gavage of *Eimeria* spp. inoculum (1 mL of PBS) was performed on D 15. Experimental groups were compared using PROC MIXED followed by the Tukey’s HSD (honestly significant difference) test. Different letters in the same row means significant differences (*p* < 0.05) among the experimental groups. Orthogonal polynomial contrasts were conducted to see linear and quadratic patterns among the experimental groups. ^2^ Standard errors of the means.

**Table 9:** Effects of different *Eimeria* infection doses on intestinal morphology parameters including villus height (VH, μm), crypt depth (CD, μm), and VH:CD in the duodenum, jejunum, and ceca of broilers on D 21 and D 35 ^1^.

	CON	ED1	ED2	ED3	ED4	SEM ^2^	*p* Value	Linear	Quadratic
D 21									
Duodenal VH	2480.12 ^a^	2381.18 ^a^	2067.76 ^ab^	1790.54 ^b^	1814.41 ^b^	248.14	<0.001	<0.001	0.464
Duodenal CD	228.78 ^d^	295.56 ^cd^	380.47 ^bc^	474 ^ab^	509.76 ^a^	66.48	<0.001	<0.001	0.603
Duodenal VH:CD	11.85 ^a^	8.32 ^b^	5.76 ^bc^	3.88 ^c^	3.88 ^c^	1.601	<0.001	<0.001	0.004
Jejunal VH	1014.28 ^a^	831.82 ^ab^	733.49 ^bc^	599.69 ^c^	553.8 ^c^	120.69	<0.001	<0.001	0.209
Jejunal CD	191.18 ^b^	359.39 ^a^	385.2 ^a^	454.07 ^a^	444.97 ^a^	65.84	<0.001	<0.001	0.005
Jejunal VH:CD	5.45 ^a^	2.45 ^b^	2.08 ^bc^	1.40 ^c^	1.29 ^c^	0.575	<0.001	<0.001	<0.001
Cecal CD	292.8 ^b^	381.5 ^ab^	502.3 ^ab^	581.3 ^a^	583.0 ^a^	155.41	0.011	<0.001	0.372
D 35									
Duodenal VH	2432.73	2319.83	2387.54	2596.8	2541.9	299.81	0.507	0.212	0.579
Duodenal CD	270.09 ^b^	358.24 ^ab^	393.32 ^a^	313.91 ^ab^	298.64 ^ab^	71.07	0.045	0.89	0.007
Duodenal VH:CD	9.50	6.72	6.63	9.05	8.97	1.91	0.032	0.609	0.012
Jejunal VH	1352.43	1358.23	1232.07	1237.21	1313.67	368.98	0.955	0.68	0.633
Jejunal CD	211.67	286.91	251.41	251.31	249.53	76.7	0.585	0.689	0.321
Jejunal VH:CD	6.86	5.33	5.25	5.30	5.51	1.44	0.278	0.154	0.113
Cecal CD	348.9	432.7	385.7	423.0	386.6	66.88	0.257	0.468	0.15

^1^ CON (unchallenged control); ED1 (*Eimeria* dose 1): *E. acervulina*: 31,250/*E. maxima*: 6250/*E. tenella*: 6250; ED2 (*Eimeria* dose 2): *E. acervulina*: 62,500/*E. maxima*: 12,500/*E. tenella*: 12,500; ED3 (*Eimeria* dose 3): *E. acervulina*: 125,000/*E. maxima*: 25,000/*E. tenella*: 25,000; ED4 (*Eimeria* dose 4): *E. acervulina*: 250,000/*E. maxima*: 50,000/*E. tenella*: 50,000. Oral gavage of *Eimeria* spp. inoculum (1 mL of PBS) was performed on D 15. Experimental groups were compared using PROC MIXED followed by the Tukey’s HSD (honestly significant difference) test. Different letters in the same row means significant differences (*p* < 0.05) among the experimental groups. Orthogonal polynomial contrasts were conducted to see linear and quadratic patterns among the experimental groups. ^2^ Standard errors of the means.

**Table 10:** Effects of different *Eimeria* infection doses on activities of jejunal brush border digestive enzymes including maltase (nmol glucose released/mg protein/min), sucrase (nmol glucose released/mg protein/min), aminopeptidase N (APN; nmol p-nitroaniline liberated/mg protein/min), lipase (mmol p-nitrophenyl phosphate liberated/mg protein/min), and intestinal alkaline phosphatase (IAP; μmol p-nitrophenol liberated/mg protein/min) and serum alkaline phosphatase (SAP; μmol p-nitrophenol liberated/mL serum/min)] in broilers on D 21 and D 35 ^1^.

	CON	ED1	ED2	ED3	ED4	SEM ^2^	*p* Value	Linear	Quadratic
D 21									
APN	12.95 ^a^	10.83 ^b^	12.71 ^ab^	11.49 ^ab^	11.46 ^ab^	1.20	0.023	0.144	0.559
Lipase	0.702	0.481	0.545	0.499	0.539	0.199	0.358	0.245	0.189
Maltase	3.508 ^a^	2.254 ^b^	3.149 ^ab^	2.297 ^ab^	2.007 ^b^	0.728	0.006	0.004	0.872
Sucrase	0.521 ^ab^	0.496 ^ab^	0.657 ^a^	0.399 ^b^	0.483 ^ab^	0.118	0.016	0.273	0.275
SAP	2.575 ^a^	1.842 ^ab^	2.08 ^ab^	1.583 ^b^	1.528 ^b^	0.544	0.017	0.003	0.463
D 35									
APN	14.54	14.37	12.37	13.97	12.20	1.67	0.054	0.027	0.880
Lipase	0.734	0.549	0.555	0.638	0.61	0.152	0.229	0.420	0.101
Maltase	4.300	3.022	3.574	3.761	3.269	1.294	0.500	0.435	0.546
Sucrase	0.499	0.394	0.524	0.453	0.457	0.206	0.844	0.927	0.962
IAP	0.157	0.13	0.16	0.149	0.163	0.029	0.328	0.445	0.358
SAP	1.536	1.506	1.734	1.639	1.465	0.414	0.795	0.989	0.345

^1^ CON (unchallenged control); ED1 (*Eimeria* dose 1): *E. acervulina*: 31,250/*E. maxima*: 6250/*E. tenella*: 6250; ED2 (*Eimeria* dose 2): *E. acervulina*: 62,500/*E. maxima*: 12,500/*E. tenella*: 12,500; ED3 (*Eimeria* dose 3): *E. acervulina*: 125,000/*E. maxima*: 25,000/*E. tenella*: 25,000; ED4 (*Eimeria* dose 4): *E. acervulina*: 250,000/*E. maxima*: 50,000/*E. tenella*: 50,000. Oral gavage of *Eimeria* spp. inoculum (1 mL of PBS) was performed on D 15. Experimental groups were compared using PROC MIXED followed by the Tukey’s HSD (honestly significant difference) test. Different letters in the same row means significant differences (*p* < 0.05) among the experimental groups. Orthogonal polynomial contrasts were conducted to see linear and quadratic patterns among the experimental groups. ^2^ Standard errors of the means.

**Table 11:** Effects of different *Eimeria* infection doses on alpha diversity parameters of the cecal microbial communities in broilers on D 21 and D 35 ^1^.

	CON	ED1	ED2	ED3	ED4	SEM ^2^	*p* Value	Linear	Quadratic
D 21									
Pielou evenness	0.610	0.646	0.575	0.586	0.634	0.108	0.756	0.923	0.526
Faith phylogenetic diversity	16.91	15.29	18.11	16.51	16.07	3.037	0.592	0.907	0.659
Shannon entropy	5.015	5.208	4.694	4.880	5.205	0.957	0.864	0.966	0.516
Observed features	298.8	267.3	303.0	316.7	295.5	56.93	0.663	0.567	0.988
D 35									
Pielou evenness	0.680	0.743	0.671	0.631	0.625	0.076	0.09	0.034	0.377
Faith phylogenetic diversity	20.5	19.26	20.15	17.31	18.12	2.338	0.129	0.036	0.914
Shannon entropy	5.94	6.506	5.656	5.328	5.259	0.752	0.051	0.015	0.520
Observed features	424.5	435.3	346.3	351.0	340.5	63.51	0.03	0.005	0.604

^1^ CON (unchallenged control); ED1 (*Eimeria* dose 1): *E. acervulina*: 31,250/*E. maxima*: 6250/*E. tenella*: 6250; ED2 (*Eimeria* dose 2): *E. acervulina*: 62,500/*E. maxima*: 12,500/*E. tenella*: 12,500; ED3 (*Eimeria* dose 3): *E. acervulina*: 125,000/*E. maxima*: 25,000/*E. tenella*: 25,000; ED4 (*Eimeria* dose 4): *E. acervulina*: 250,000/*E. maxima*: 50,000/*E. tenella*: 50,000. Oral gavage of *Eimeria* spp. inoculum (1 mL of PBS) was performed on D 15. Experimental groups were compared using PROC MIXED followed by the Tukey’s HSD (honestly significant difference) test. Orthogonal polynomial contrasts were conducted to see linear and quadratic patterns among the experimental groups. ^2^ Standard errors of the means.

**Table 12:** Effects of different doses of *Eimeria* spp. on the relative mRNA expression of genes relating to viability and sexual reproduction of *E. acervulina*, *E. maxima*, and *E. tenella* in broilers on D 21 ^1,2^.

	ED1	ED2	ED3	ED4	SEM ^3^	*p* Value	Linear	Quadratic
*E. acervulina*								
*18s*	1.023	4.913	1.245	3.918	2.457	0.048	0.277	0.551
*APN*	1.038	1.058	1.238	1.038	0.824	0.969	0.908	0.753
Flagella-related protein	1.056	1.295	1.841	1.066	1.215	0.664	0.806	0.331
*EF1*	1.04	0.947	1.83	1.068	0.715	0.277	0.556	0.356
*EF2*	1.008	0.957	1.47	0.857	0.721	0.483	0.965	0.363
*GAM56*	1.022	0.889	2.36	1.1	1.559	0.358	0.573	0.398
*GAM82*	1.010	0.950	1.238	1.067	0.734	0.916	0.748	0.858
*E. maxima*								
*18s*	1.444	1.370	0.652	0.592	1.062	0.373	0.107	0.989
*APN*	1.059 ^b^	1.096 ^b^	2.151 ^a^	1.775 ^ab^	0.62	0.015	0.01	0.424
Flagella-related protein	1.044	0.972	1.041	0.92	0.339	0.904	0.628	0.861
*EF1A*	1.027	1.355	0.973	1.199	0.477	0.51	0.879	0.796
*EF2*	1.024	1.225	1.481	1.335	0.308	0.104	0.048	0.184
*GAM56*	1.035 ^c^	1.241 ^bc^	2.022 ^ab^	2.144 ^a^	0.534	0.003	<0.001	0.85
*GAM82*	1.021 ^b^	1.102 ^ab^	1.819 ^a^	1.595 ^ab^	0.457	0.017	0.008	0.422
*E. tenella*								
*18s*	4.11	68.12	58.98	27.34	77.12	0.401	0.646	0.115
*APN*	2.361	0.743	0.598	1.548	1.954	0.44	0.496	0.133
Flagella-related protein	1.310	1.904	1.048	1.852	1.259	0.610	0.752	0.848
*EF1A*	1.326	1.534	1.267	0.979	1.055	0.838	0.524	0.581
*GAM22*	1.218	1.087	1.007	1.852	1.092	0.542	0.393	0.299
*GAM56*	3.682	2.623	1.868	6.219	4.912	0.461	0.473	0.204

^1^ CON (unchallenged control); ED1 (*Eimeria* dose 1): *E. acervulina*: 31,250/*E. maxima*: 6250/*E. tenella*: 6250; ED2 (*Eimeria* dose 2): *E. acervulina*: 62,500/*E. maxima*: 12,500/*E. tenella*: 12,500; ED3 (*Eimeria* dose 3): *E. acervulina*: 125,000/*E. maxima*: 25,000/*E. tenella*: 25,000; ED4 (*Eimeria* dose 4): *E. acervulina*: 250,000/*E. maxima*: 50,000/*E. tenella*: 50,000. Oral gavage of *Eimeria* spp. inoculum (1 mL of PBS) was performed on D 15. Experimental groups were compared using PROC MIXED followed by the Tukey’s HSD (honestly significant difference) test. Different letters in the same row means significant differences (*p* < 0.05) among the experimental groups. Orthogonal polynomial contrasts were conducted to see linear and quadratic patterns among the experimental groups. ^2^ Relative abundance of *Eimeria* 18s genes was normalized by using host reference genes (beta actin and glyceraldehyde 3-phosphate dehydrogenase), and relative abundance of *Eimeria* genes including aminopeptidase N *(APN)*, elongation factor *(EF)*, and gametocyte protein *(GAM)* were normalized by using *Eimeria* 18s genes. ^3^ Standard errors of the means.

**Table 13:** Effects of different *Eimeria* infection doses on body composition parameters including total weight (kg), fat (kg), lean weight (kg), fat percentage (%), lean percentage (%), and lean:fat in broilers ^1^.

	CON	ED1	ED2	ED3	ED4	SEM ^2^	*p* Value	Linear	Quadratic
D 21									
Total weight	0.825 ^a^	0.737 ^ab^	0.719 ^ab^	0.709 ^ab^	0.651 ^b^	0.086	0.028	0.002	0.609
Fat	0.119	0.094	0.098	0.091	0.084	0.024	0.144	0.023	0.532
Lean weight	0.706 ^a^	0.643 ^ab^	0.620 ^ab^	0.640 ^ab^	0.567 ^b^	0.071	0.038	0.005	0.838
Fat percentage	14.28	12.78	13.63	12.68	12.80	2.22	0.668	0.294	0.676
Lean percentage	85.71	87.22	86.37	87.34	87.20	2.345	0.657	0.287	0.661
Lean:fat	6.138	6.873	6.721	7.359	7.043	1.512	0.711	0.251	0.575
D 28									
Total weight	1.309	1.224	1.259	1.295	1.272	0.13	0.823	0.988	0.538
Fat	0.176	0.162	0.147	0.160	0.153	0.04	0.788	0.379	0.494
Lean weight	1.134	1.062	1.113	1.135	1.119	0.114	0.806	0.767	0.641
Fat percentage	13.37	13.02	11.70	12.32	12.08	2.73	0.825	0.364	0.608
Lean percentage	86.64	86.99	88.29	87.67	87.89	2.74	0.834	0.377	0.609
Lean:fat	6.553	7.289	8.063	7.670	7.462	2.174	0.81	0.441	0.366
D 35									
Total weight	2.19	1.70	2.04	2.03	1.90	0.37	0.238	0.603	0.497
Fat	0.382 ^a^	0.346 ^ab^	0.338 ^ab^	0.342 ^ab^	0.297 ^b^	0.04	0.055	0.006	0.925
Lean weight	1.810	1.616	1.699	1.684	1.606	0.147	0.107	0.068	0.532
Fat percentage	17.40	17.67	16.60	16.85	15.60	1.72	0.293	0.060	0.52
Lean percentage	82.61	82.30	83.41	83.15	84.39	1.72	0.291	0.059	0.513
Lean:fat	4.772	4.671	5.051	5.048	5.540	0.695	0.258	0.043	0.455

^1^ CON (unchallenged control); ED1 (*Eimeria* dose 1): *E. acervulina*: 31,250/*E. maxima*: 6250/*E. tenella*: 6250; ED2 (*Eimeria* dose 2): *E. acervulina*: 62,500/*E. maxima*: 12,500/*E. tenella*: 12,500; ED3 (*Eimeria* dose 3): *E. acervulina*: 125,000/*E. maxima*: 25,000/*E. tenella*: 25,000; ED4 (*Eimeria* dose 4): *E. acervulina*: 250,000/*E. maxima*: 50,000/*E. tenella*: 50,000. Oral gavage of *Eimeria* spp. inoculum (1 mL of PBS) was performed on D 15. Experimental groups were compared using PROC MIXED followed by the Tukey’s HSD (honestly significant difference) test. Different letters in the same row means significant differences (*p* < 0.05) among the experimental groups. Orthogonal polynomial contrasts were conducted to see linear and quadratic patterns among the experimental groups. ^2^ Standard errors of the means.

## Data Availability

Data are unavailable due to privacy restrictions.
